# Apatinib in patients with extensive-stage small-cell lung cancer after second-line or third-line chemotherapy: a phase II, single-arm, multicentre, prospective study

**DOI:** 10.1038/s41416-019-0583-6

**Published:** 2019-09-16

**Authors:** Yanjun Xu, Zhiyu Huang, Hongyang Lu, Xinming Yu, Yuping Li, Wenfeng Li, Jun Chen, Ming Chen, Lei Gong, Kaiyan Chen, Jin Qin, Xiaoling Xu, Ying Jin, Jun Zhao, Xun Shi, Na Han, Fajun Xie, Peng Zhang, Weizhen Xu, Yun Fan

**Affiliations:** 1 Department of Medical Thoracic Oncology, Zhejiang Cancer Hospital, No. 1 Banshan East Road, Hangzhou, China; 2 Department of Pulmonary and Critical Care Medicine, The First Affiliated Hospital of Wenzhou Medical University, Wenzhou, China; 3 Department of Radiotherapy and Chemotherapy, Yinzhou People’s Hospital, Ningbo, China; 4 Department of Radiation Oncology, Zhejiang Cancer Hospital, Hangzhou, China; 5 Good Clinical Practice Center of Zhejiang Cancer Hospital, Hangzhou, China; 6 Key laboratory on Diagnosis and Treatment Technology on Thoracic Cancer, Zhejiang Cancer Hospital (Zhejiang Cancer Research Institute), Hangzhou, China

**Keywords:** Targeted therapies, Small-cell lung cancer

## Abstract

**Background:**

Small-cell lung cancer (SCLC) remains an aggressive cancer with short-term survival due to limited therapeutic options. Apatinib is a small-molecule tyrosine kinase inhibitor that selectively inhibits vascular endothelial growth factor receptor-2. This study aimed to investigate the efficacy and safety of apatinib in patients with extensive-stage (EC) SCLC who had progressed after two or three previous therapies.

**Methods:**

Eligible patients were histologically confirmed ES-SCLC after two or three previous treatments, including a platinum-based regimen. Patients received apatinib at an initial dose of 500 mg once daily. The primary endpoint was the objective response rate.

**Results:**

Forty patients were enrolled. At the data cut-off time (November 15, 2018), the median follow-up was 7.4 months; no patients remained on treatment, and five were still in follow-up. An objective response was achieved in 7 of 40 patients (17.5%) in the intention-to-treat population, and 7 of 38 patients (18.4%) in the per-protocol population. The median progression-free survival and overall survival were 3.0 months and 5·8 months, respectively. The most commonly observed grade 3 or greater treatment-related adverse events were hypertension, hand–foot syndrome, increased L-gamma-glutamyltransferase.

**Conclusions:**

Apatinib exhibited efficacy and an acceptable safety profile in previously heavily-treated ES-SCLC patients. Further exploration of apatinib in phase III trials is warranted.

**Trial registration:**

NCT02945852.

## Background

Small-cell lung cancer (SCLC) accounts for ~15% of all lung cancers^1^ and is biologically characterised by high aggressiveness and early widespread metastasis, resulting in the majority of patients being diagnosed with extensive-stage disease (ES-SCLC).^2,3^ Therapeutic strategies have not substantially changed in >40 years, and the long-term survival rate remains dismal. Overall, the median survival for ES-SCLC is 9–11 months, and the 5-year survival rate is only ~2%.^4,5^ Despite a high response rate with initial platinum-based chemotherapy, almost all patients with ES-SCLC will subsequently relapse after a short period of response.^3–7^ Consequently, therapeutic options are limited, and there is an especially poor prognosis when a recurrence occurs. Topotecan is the only approved standard second-line treatment. The reported response rate of topotecan ranged from 5 to 17% in SCLC patients after first-line chemotherapy.^8^ After failure of first- or second-line treatment, nivolumab was recently approved for third-line setting of ES-SCLC by the US Food and Drug Administration, and nivolumab alone or in combination with ipilimumab was recommended for second-line systemic therapy in National Comprehensive Cancer Network guidelines. Nevertheless, monotherapy with both topotecan and nivolumab is challenging due to their modest anti-tumour activities.^8,9^ There is still an unmet need for novel agents for the treatment of patients with ES-SCLC who have progressed after two or more previous treatment regimens.

Angiogenesis plays a pivotal role in tumour growth, progression and metastasis of SCLC.^10^ Preclinical studies have indicated that SCLC can highly express the VEGF receptors (e.g., VEGFR2 and VEGFR3).^11,12^ Blockade of the interaction between VEGF and VEGFRs can effectively inhibit SCLC growth.^13,14^ The currently available anti-angiogenic agents include VEGF monoclonal antibodies (e.g., bevacizumab) and small molecule, multitargeted tyrosine kinase inhibitors (TKIs), which target the VEGFRs (e.g., sunitinib, sorafenib, vandetanib, etc.). Several clinical trials have reported encouraging results with anti-angiogenic agents in some settings of SCLC.^15–18^ For example, addition of bevacizumab to chemotherapy for previously untreated ES-SCLC patients demonstrated a significant prolongation of progression-free survival than standard chemotherapy (6.7 vs 5.7 months; *p* = 0.030).^18^ A phase II study reported that sunitinib as maintenance therapy yielded a longer PFS for ES-SCLC patients after first-line chemotherapy (3.7 vs 2.1 months; *p* = 0.02).^19^ However, both of them failed to extend the overall survival. Of note, a recent phase III trial (IMpower133) demonstrated a significantly longer progression-free survival, and overall survival with atezolizumab plus etoposide and carboplatin than with placebo plus etoposide and carboplatin.^20^ These findings suggested that anti-angiogenic treatment is feasible in this population, but novel agents and combinational strategies are still needed for further investigation.^15–19^

Apatinib is a novel oral, small-molecule TKI that selectively targets VEGFR2. Previous studies have demonstrated that apatinib has encouraging anti-tumour activity with tolerable toxicity in several types of solid tumours, including SCLC.^21^ Apatinib has been approved for the treatment of advanced or metastatic chemorefractory gastric cancer in China.^22,23^ To date, two retrospective studies in China have evaluated apatinib as third-line to fifth-line therapy and as maintenance therapy in ES-SCLC, respectively. These studies suggested that apatinib has promising activity and acceptable toxicity in ES-SCLC.^24^ Therefore, we conducted this phase II, single-arm, multicentre study to prospectively investigate the efficacy and safety of apatinib in Chinese patients with ES-SCLC who had experienced disease progression after second-line or third-line chemotherapy.

## Methods

### Study design and participants

Patients with ES-SCLC were enrolled in a prospective multicentre, open-label, single-arm, phase II trial at three medical centres in China (Zhejiang Cancer Hospital, Ningbo Yinzhou People’s Hospital, and The First Affiliated Hospital of Wenzhou Medical University). Eligible patients had histologically confirmed ES-SCLC, with disease progression (according to the Response Evaluation Criteria in Solid Tumors [RECIST], version 1.1) after two or three previous chemotherapy treatments, including a platinum-based regimen. Both platinum-sensitive patients (relapse ≥ 90 days after chemotherapy) and platinum-resistant patients (relapse < 90 days after or during chemotherapy) were eligible. Patients were 18 years of age or older, with an Eastern Cooperative Oncology Group (ECOG) performance status of 0–2, and a life expectancy of at least 12 weeks, with one or more measurable tumour lesions (according to RECIST, version 1.1). Patients with a history of treated central nervous system (CNS) metastases were eligible as there was no ongoing requirement for corticosteroids as therapy for asymptomatic CNS disease. Other inclusion criteria were: (1) adequate bone marrow function (white blood cell count of ≥ 3.5 × 10^3^ cells/μL, an absolute neutrophil count of ≥ 1.5 × 10^3^ cells/μL, a platelet count of ≥ 100 × 10^3^ cells/μL and a haemoglobin concentration of ≥ 9.0 g/dL); and (2) adequate hepatic function (aspartate transaminase and alanine transaminase ≤ 1.5 × upper limit of normal, bilirubin level ≤ 1.5 × upper limit of normal) and renal function.

Key exclusion criteria were mixed SCLC, previous exposure to apatinib or other anti-angiogenic agents, active or new diagnosed untreated CNS metastases, uncontrolled hypertension, major surgical procedures within 4 weeks before treatment initiation and/or the presence of any non-healing wound, fracture, ulcer, evidence of active bleeding or significant haemoptysis (≥ 5 mL). Other exclusion criteria included significant cardiac morbidity, a history of cerebrovascular accident, transient ischaemic attack, pulmonary embolism or untreated deep venous thrombosis within the past 6 months, severe clinical infections, any other life-threatening illnesses and other malignancies diagnosed within the past 5 years other than non-melanoma skin cancer.

The study protocol was approved by the relevant institutional review board or ethics committee at each medical centre, and it was conducted in accordance with the Declaration of Helsinki and Good Clinical Practice guidelines, as defined by the International Conference on Harmonisation. All enrolled patients provided written informed consent before any study-specific procedures were performed.

### Procedures

Apatinib was administered orally at an initial dose of 500 mg once daily, continuously in 30-day cycles. Treatment was continued until disease progression, patient withdrawal, unacceptable toxicity or death occurred. Dose interruptions or reductions were permitted for management of adverse events (AEs), but only one dose reduction per patient was allowed (250 mg once daily) and dose re-escalation was not permitted. When ≥ grade 3 haematological or ≥ grade 2 non-haematological toxicities or a clinically intolerable grade 2 AEs occurred at any time, dose interruptions or reductions were allowed. In such cases, treatment would be resumed at a reduced dosage of 250 mg once daily after recovery to ≤ grade 2 haematological or ≤ grade 1 non-haematological toxicity. Repeated dose interruptions were allowed for a maximum of 14 days on each occasion. Once dose interruption had occurred, subsequent apatinib was resumed at a reduced dosage of 250 mg once daily.

Tumour assessments were conducted by radiographic imaging [computed tomography (CT) and magnetic resonance imaging (MRI)] at baseline, after the first cycle of apatinib treatment, and then every 2 months (± 7 days) thereafter until disease progression occurred (investigator assessed per RECIST, version 1.1) or treatment was discontinued. Once a partial response was occurred in the evaluation of apatinib efficiency, another CT scan will be added 1 month later to confirm the partial response. If clinical symptoms of patients aggravate, we would consider taking CT scan ahead of time. Baseline tumour assessments included at least enhanced CT of the chest, abdomen and pelvis, and enhanced MRI of the brain. Repeated radiographic imaging included enhanced CT of the chest and abdomen, and enhanced MRI of the brain in cases of symptom occurrence. Survival was monitored continuously during treatment, and every 2 months after its discontinuation. Haematology, serum chemistry, routine urine examinations, vital signs, physical examinations and 12 lead electrocardiograms were assessed every month during treatment.

A safety evaluation was performed throughout the study, during treatment and within 30 days of administration of the last dose of apatinib.

### Outcomes

The primary endpoint was the proportion of patients with a confirmed complete or partial response as per RECIST evaluation, version 1.1. Secondary endpoints included progression-free survival, overall survival, disease control rate and the occurrence of treatment-related AEs. Progression-free survival was defined as the time from treatment assignment to the date of the first objectively documented disease progression, or death due to any cause, whichever occurred first. Overall survival was defined as the time from the date of treatment assignment to the date of death due to any cause. The disease control rate was defined as the percentage of patients who achieved a complete response, partial response or stable disease per RECIST evaluation, version 1.1. Treatment-related AEs were graded according to the National Cancer Institute’s Common Terminology Criteria for Adverse Events (NCI-CTCAE, version 4.03).

### Statistical analysis

This study followed Simon’s two-stage optimum design, with a type I error rate of 10% and a power of 80% to reject the null hypothesis.^25^ The previously reported objective response rate with topotecan monotherapy as second-line therapy in SCLC is ~7%.^26^ In this study, the primary expectation of an objective response with apatinib monotherapy was 19%. Consequently, 18 eligible patients received treatment in the first stage of the study with at least two responses required to continue the enrolment. In stage 2, 19 additional patients were enrolled for a total sample size of 37. Overall, if a total of five responses or more were observed, the treatment regimen would be considered successful. Assuming a 5% missing follow-up rate for the subjects, a total sample size of 39 was required. This study design planned to enrol 40 patients.

The following three populations were analysed: the full analysis set (FAS), the per-protocol set (PPS) and the safety analysis set (SAS). The FAS consisted of all enrolled patients who received at least one dose of study medication according to the intention-to-treat (ITT) principle. The PPS referred to a subset of the patients in the FAS who were compliant with the protocol and without any major protocol violations (including violation of study entry criteria). The SAS included all enrolled patients who received at least one dose of the study medication, though not those without any safety data. Patients’ baseline characteristics were summarised for the ITT population. The efficacy analysis was performed in both the ITT and PPS populations. Safety data were explored in the SAS.

Objective response and disease control rate were evaluated via the binomial response rate and the corresponding two-sided 95% exact confidence intervals (CIs), using the Clopper–Pearson method. The Kaplan–Meier method was used to estimate progression-free survival and overall survival, and median values were estimated with two-sided 95% CIs by using the Brookmeyer–Crowley method. Cox proportional-hazards regression models were used for univariate and multivariate analysis to identify the potential factors associated with clinical outcomes. A *p*-value (two-sided) < 0.05 was considered statistically significant. All analyses were performed with SPSS^®^ 22.0. The study was registered with ClinicalTrials.gov (NCT02945852).

## Results

### Patient demographics

**Fig. 1:**
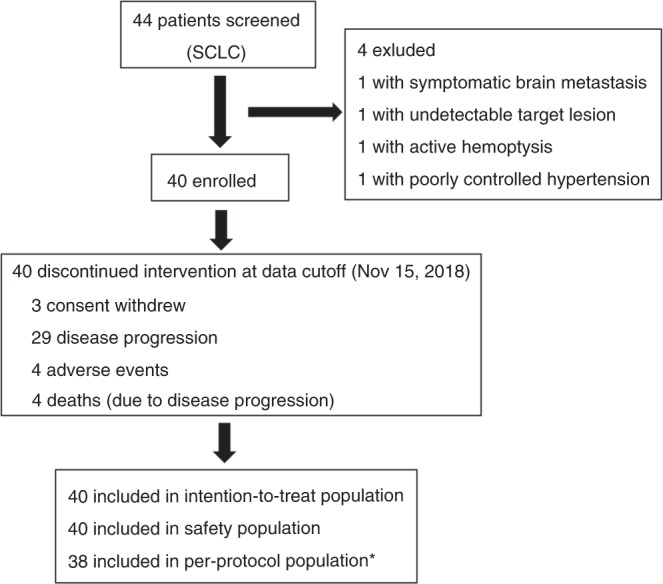
Trial profile. ^*^Two patients were excluded because they had no post-baseline efficacy assessment

Between July 10, 2016 and April 30, 2018, a total of 44 patients with ES-SCLC were screened at three medical centres, and 40 of these patients were enrolled in the study (Fig. 1). All received at least one dose of apatinib. The exclusions were mainly due to the following reasons: symptomatic brain metastasis, undetectable target lesions, active haemoptysis and poorly controlled hypertension. The baseline characteristics of the 40 enrolled patients are shown in Table 1. Their median age was 60 years (range, 39–71 years), and 37 (92.5%) were male. Four patients (10%) had a never-smoking history, and 17.5 and 60.0% presented brain and liver metastases, respectively. All patients had received prior chemotherapy for ES-SCLC; 31 patients (77.5%) had received two prior lines of chemotherapy and 9 patients (22.5%) had received three prior lines of chemotherapy. The best response to the most recent chemotherapy treatment was stable disease.

**Table 1:** Baseline patient characteristics

	Variable	Apatinib (*n* = 40)
Median age (years)		60 (39–71)
Gender	Male	37 (92.5)
	Female	3 (7.5)
ECOG performance status	0	1 (2.5)
	1	38 (95.0)
	2	1 (2.5)
Smoking status	Never	4 (10)
	Former/current	36 (90)
Stage (VALG)	Limited	0 (0.0)
	Extensive	40 (100.0)
Brain metastases	No	33 (82.5)
	Yes	7 (17.5)
Liver metastases	No	16 (40.0)
	Yes	24 (60.0)
Previous lines of treatment	2	31 (77.5)
	3	9 (22.5)
Relapse type	Refractory	17 (42.5)
	Sensitive	23 (57.5)
Platinum re-challenge in second-line treatment	Yes	27 (67.5)
	No	13 (22.5)

*VALG* Veterans Administration Lung Study Group, *ECOG* Eastern Cooperative Oncology Group

The data presented as *n* (%) or median (IQR) unless otherwise specified

### Apatinib treatment

At the time of data cut-off (November 15, 2018), the median follow-up duration was 7.4 months (IQR 6.6; range, 1.0–22.2 months). No patients remained on active treatment at this time, but five (12.5%) remained in follow-up. The most common reason for treatment discontinuation was disease progression (29 patients [72.5%]) (Fig. 1). Other reasons for treatment discontinuation included disease progression-related death (four patients [10.0%]), AEs (four [10.0%]) and withdrawal of consent (three [7.5%]). Fifteen of the 40 patients (37.5%) required apatinib dosage reduction. Among them, eight had reduction for apatinib during or at the completion of the first treatment cycle, the occurrence of dose reduction was recorded in six patients during the second cycle, and in one patient during the fifth cycle. At the data cut-off point, 40 patients received apatinib 500 mg once daily with a median of 1.7 months (IQR 1.9; range 0.3–10.2), and 15 patients received apatinib 250 mg once daily for a median of 1.9 months (IQR 2.3; range 0.2–10.6). As mentioned above, dose reductions occurred in 15 of 40 patients for apatinib, and all the 15 patients required only one dose reduction. Among the 15 patients, two experienced drug withdrawals prior to dose reduction.

### Efficacy

**Fig. 2:**
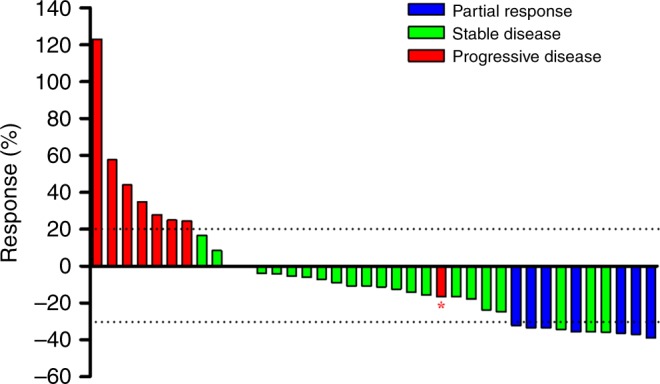
Waterfall plot for the best percentage change in target lesion size (*n* = 38). Waterfall plot for the best percentage change in target lesion size is shown for 38 patients who had at least one post-baseline efficacy assessment. The colours indicate type of responses. The dashed line at 20% represents the boundary for determination of progressive disease, and the dashed line at –30% represents the boundary for determination of partial response. *Tumour shrinkage over 30% was observed in this patient, but there were new lesions, so it was judged to be disease progression

The predefined threshold of two or more responses for the first stage of Simon’s two-stage optimum design was met, as four (22.2%) of the first 18 evaluable patients achieved objective responses. Therefore, the enrolment was continued to full accrual. No complete responses were observed, but partial responses were achieved in seven of the 40 patients (17.5%) in the ITT population and in seven of the 38 patients (18.4%) in the per-protocol population (Table 2). Disease control was achieved in 30 patients (75.0%; 61–89) in the ITT population, and in 30 patients (78.9%; 65.4–92.5) in the per-protocol population (Table 2). Disease control rate at 3 months was and 42.5% (95% CI, 27.0–59.1%) and 7.5% (95% CI, 1.6–20.4%) at 6 months. Tumour shrinkage was observed in 27 of the 38 patients (71.1%) who had at least one post-baseline efficacy assessment (Fig. 2).

**Table 2:** Treatment responses

	Intention-to-treat population (*n* = 40)	Per-protocol population (*n* = 38)^a^
Complete response	0	0
Partial response	7 (17.5%)	7 (18.4%)
Stable disease	23 (57.5%)	23 (60.5%)
Disease progression	8 (20.0%)	8 (21.1%)
Overall response	7 (17.5%; 5.2–29.8)	7 (18.4%; 5.5–31.3)
Disease control	30 (75.0%; 61–89)	30 (78.9%; 65.4–92.5)

The data presented as *n* (%)

^a^Two patients were excluded because there was no post-baseline efficacy assessment

**Fig. 3:**
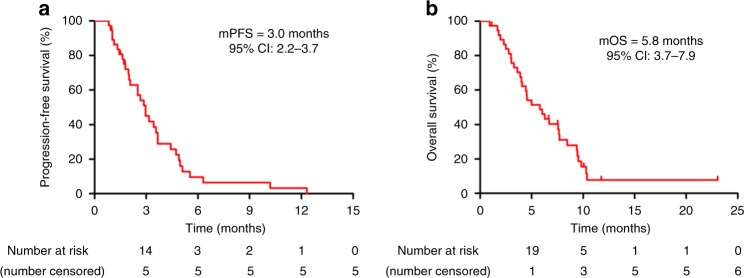
Kaplan–Meier estimates for progression-free survival (**a**) and overall survival (**b**) in patients with at least one post-baseline efficacy assessment (*n* = 38)

All of the seven patients who had objective responses experienced disease progression or had died by the data cut-off point. By the data cut-off date, a total of 25 patients (62.5%) had died. All 13 patients who were still alive at this time had discontinued apatinib treatment, 11 of whom had switched to further treatment, including gemcitabine or docetaxel regimens. The median progression-free survival and overall survival were 3.0 months (95% CI 2.2–3.7; Fig. 3a) and 5·8 months (95% CI 3.7–7.9; Fig. 3b), respectively. The 6-months OS rate was 48.7% (95% CI 32.6–64.8).

### Safety

The SAS included the entire 40 patients, and all experienced AEs related to apatinib treatment. AEs of clinical significance, as selected by the investigators, are summarised in Table 3. The most commonly observed grade 3 or greater AEs were hypertension (25%; 10/40), hand–foot syndrome (10%; 4/40), increased L-gamma glutamyltransferase (10%; 4/40), increased aspartate transaminase (7.5%; 3/40) and thrombocytopenia (7.5%; 3/40). In addition, one patient required hospitalisation for the management of grade 4 neutropenia. No grade 5 AEs were reported, and no unexpected AEs were observed during the study.

**Table 3:** All adverse events associated with apatinib treatment of any grade in the safety population (*n* = 40)

*n* (%)	Grades 1–2	Grade 3	Grade 4	Grade 5
*Non-haematological*				
Hypertension	17 (42.5%)	10 (25%)	0 (0%)	0 (0%)
Proteinuria	30 (75%)	0 (0%)	–	–
Hand–foot syndrome	23 (57.5%)	4 (10%)	–	–
Mucositis	7 (17.5%)	2 (5%)	0 (0%)	0 (0%)
Haematuria	18 (45%)	0 (0%)	0 (0%)	–
Headache	11 (27.5%)	0 (0%)	–	–
Nausea	10 (25%)	0 (0%)	–	–
Vomit	8 (20%)	0 (0%)	0 (0%)	0 (0%)
Diarrhoea	8 (20%)	0 (0%)	0 (0%)	0 (0%)
Fatigue	23 (57.5%)	0 (0%)	–	**–**
Increased ALT	14 (35%)	0 (0%)	0 (0%)	–
Increased AST	24 (60%)	3 (7.5%)	0 (0%)	–
Increased alkaline phosphatase	19 (47.5%)	0 (0%)	0 (0%)	–
Increased L-gamma glutamyltransferase	18 (45%)	3 (7.5%)	1 (2.5%)	–
Urobilinogen	17 (42.5%)	0 (0%)	0 (0%)	–
Increased creatinine	8 (20%)	0 (0%)	0 (0%)	–
*Haematological*				
Neutropenia	9 (22.5%)	1 (2.5%)	1 (2.5%)	0 (0%)
Anaemia	32 (80%)	0 (0%)	0 (0%)	0 (0%)
Thrombocytopenia	16 (40%)	3 (7.5%)	0 (0%)	0 (0%)

Dosage reduction of apatinib was required in 15 patients (37.5%) during the therapy with following reasons: 11 cases (73.3%) of hand–foot syndrome, 10 (66.7%) cases of hypertension, one (6.7%) case of proteinuria one (6.7%) case of mucositis, one (6.7%) case of thrombocytopenia and one (6.7%) case of impaired liver function. Of note, 10 of the 15 patients (66.7%) needed dosage reduction due to two (60%; *n* = 9) or three (6.7%; *n* = 1) toxic reactions. Treatment had to be stopped due to intolerable toxic reactions for four patients, one for fatigue, two cases for hand–foot syndrome and one case for proteinuria. The reasons for apatinib dose reduction are summarised in Supplementary Table 1.

### Univariate and multivariate analysis

The presence or absence of liver or brain metastases, and the prior therapy lines have been considered as the important prognostic factors in patients with ES-SCLC. These factors together with other baseline parameters were selected for univariate analysis of prognostic factors for progression-free survival and overall survival. The results are summarised in Supplementary Tables 2 and 3. Liver metastasis (HR 3.6; 95% CI 1.5–8.5; *p* = 0.004) was the only independent prognostic factor associated with a significantly shorter progression-free survival by univariate analysis (Supplementary Table 2), and it remained statistical significance in the multivariate analysis. The median progression-free survival values for patients with and without liver metastases were 2.0 months (95% CI 1.0–3.0) and 4·7 months (95% CI 1.9–7.6), respectively; *p* = 0.007; HR = 4.0 (95% CI 1.5–10.6) (Supplementary Fig. 1). No significance was found for the association of other factors with progression-free survival. Also, no factors were observed to be associated with overall survival (Supplementary Table 3). As shown in Supplementary Tables 4 and 5, no correlation was found between observed AEs and either progression-free survival or overall survival during apatinib treatment.

## Discussion

To our knowledge, this is the first prospective phase II trial to evaluate apatinib monotherapy in a third-line or above setting for patients with ES-SCLC. The results indicated that apatinib monotherapy provided encouraging efficacy with an acceptable safety profile for patients with ES-SCLC who had progressed after two or three previous regimens. Of the 38 patients able to be assessed in the efficacy analysis, the objective response rate was 18.4%, and the median progression-free survival and overall survival were 3.0 and 5.8 months, respectively. No unexpected treatment-related AEs were observed.

To date, only nivolumab was approved for third-line treatment in patients with ES-SCLC, but the efficacy is modest. The limited therapeutic options available for patients after first- and second-line chemotherapy is one of the important reason for the dismal prognosis of patients with recurrent ES-SCLC. Published studies on the efficacy of systemic therapy in the third-line plus setting are sparse, and most of them are single-centre retrospective or small single-arm trials.^27–29^ Two large-scale multicentre international retrospective studies have been reported, and all showed the effectiveness of active systemic therapy compared with best supportive care or palliative care. One of them analysed 120 patients from three countries in the third-line setting and found the median progression-free survival and overall survival were 2.0 and 4.7 months, respectively.^30^ Another study evaluating 334 SCLC patients received third-line plus systemic therapy, and reported physician-assessed objective response rate of 21%, progression-free survival of 2.3 months and overall months of 4.4 months.^31^ This study is the first prospective study in third-line plus treatment of ES-SCLC. Comparing to these two retrospective studies with the current study suggests a non-inferior objective response rate, but better progression-free survival and overall survival of apatinib monotherapy. Although direct comparisons across studies with different designs and patient populations is arbitrary, our study indicates that active systemic therapy in third-line plus setting is clinically valuable in ES-SCLC patients with good performance status. But a phase III clinical trial is still needed before apatinib could be seriously considered in this setting.

As previously mentioned, the activities of anti-angiogenic therapies have also been explored in patients with ES-SCLC from first-line to third-line or above settings. Earlier phase II studies investigating bevacizumab in combination with chemotherapy for previously untreated ES-SCLC, yielded a progression-free survival of 4.7–7.0 months, an overall survival of 9.4–11.6 months and an acceptable safety profile.^16,17^ Subsequently, a phase III study (GOIRC-AIFA FARM6PMFJM trial) provided additional supportive evidence that use of this combination for first-line treatment of ES-SCLC produced a statistically significant improvement in progression-free survival (6.7 vs 5.7 months; HR 0.72; *p* = 0.030).^18^ However, this advancement did not translate into noticeable overall survival benefit (8.9 vs 9.8 months; HR, 0.78; *p* = 0.113). In second-line setting, bevacizumab plus topotecan did not meet the predefined criteria for clinically meaningful improvement in patients with relapsed SCLC (PFS rate at 3 months was 65%).^32^ Combining aflibercept with weekly topotecan for previously treated SCLC patients was significantly associated with better 3-month PFS for patients with refractory disease, but not sensitive disease.^33^ Overall survival was still not improved. Similar to these findings, maintenance sunitinib in a phase II study of patients with ES-SCLC yielded a meaningful progression-free survival result (3.7 vs 2.1 months; HR 1.62, *p* = 0.02), but it failed to extend the overall survival (*p* = 0.16).^19^ In this study, apatinib showed an objective response rate of 18.4% in a third-line or greater setting in patients with ES-SCLC, suggesting its more significant anti-tumour effect than other anti-angiogenic agents.

Liver metastasis is one of the most common distant metastases in ES-SCLC, and it confers a very poor prognosis in these patients. In this study, liver metastasis was identified as the only independent prognostic factor for progression-free survival in the multivariate analysis. Of note, although the current cohort enrolled a higher proportion of patients with liver metastasis (60.0%) in comparison with other reports,^9,10,15–17,26^ the reported overall survival rate was non-inferior, suggesting the potential efficacy of apatinib in this population. Interestingly, a recent study reported that EGFR-TKIs plus local therapy showed prolonged survival benefit than EGFR-TKIs alone in EGFR-mutant NSCLC patients with oligometastatic or oligoprogressive liver metastases, suggesting the good control effect of local therapy in patients with liver metastases.^34^ In view of the poor response of patients with liver metastasis to apatinib monotherapy, combination of apatinib and local consolidation strategies (e.g., radiotherapy and radiofrequency ablation) should be investigated for patients with SCLC and liver metastasis.

The most commonly reported AEs of apatinib are hypertension, hand–foot syndrome, proteinuria and fatigue. This AE profile was also observed in this study with incidence rates of hypertension (67.5%), hand–foot syndrome (67.5%), proteinuria (75%) and fatigue (57.5%). It is worth noting that no grade 3 or greater fatigue was observed. All cases of proteinuria were grade 1 to 2. The incidence of grade 3 hand–foot syndrome and hypertension were 10.0 and 25.0%, respectively. Almost all AEs were improved by symptomatic treatment and/or apatinib dose reduction or interruptions in this study. Other AEs were mainly grade 1 to 2 with a low incidence rate. Only one case each of grade 4 neutropenia and grade 4 increased L-gamma-glutamyltransferase was observed. No treatment-related death was recorded. Moreover, toxicities with VEGFR-TKIs have been reported as predictive biomarkers for treatment efficacy in previous studies, but no significant relationship was observed between AEs and either progression-free survival or overall survival in this study.

Immunotherapy targeted PD-1 and its ligand (PD-L1) has shown good anti-tumour activity in various solid tumours including SCLC. Although the phase I-II trial of nivolumab alone or in combination with ipilimumab for previously treated SCLC patients (CheckMate 032) reported the modest anti-tumor effect,^9^ it was recommended by the National Comprehensive Cancer Network guideline for second-line treatment.^9^ However, two phase III trials (CheckMate 451 and CheckMate 331) that evaluated nivolumab plus ipilimumab, or nivolumab alone as maintenance therapy or second-line setting failed to meet their primary endpoints. Nevertheless, a recent phase III trial (IMpower133) demonstrated a significantly longer progression-free survival and overall survival with atezolizumab plus etoposide and carboplatin than with placebo plus etoposide and carboplatin,^20^ which has become a new standard of care in the first-line setting of ES-SCLC. These findings suggested that immunotherapy in combination with other anti-tumour strategies is promising and efficacious in ES-SCLC. Considering the synergistic effect of immunotherapy plus anti-angiogenic therapy,^35,36^ a recent study found that apatinib could potentiate the anti-tumour effect of a PD-1/PD-L1 inhibitor in the lung cancer mouse model by optimising the tumour microenvironment.^37^ In a preliminary investigation, they found an objective response rate of 55.6% in patients with NSCLC and a low incidence of PD-L1 expression. Furthermore, the synergistic anti-tumour effect of PD-1/PD-L1 inhibitors plus anti-angiogenic agents is also evident in other solid tumours, such as gastric or oesophagogastric junction cancer patients.^38^ Collectively, future combinational strategies based on PD-1/-DL1 antibodies in ED-SCLC could be relevant and of clinical interest. We have therefore initiated a phase II study to investigate SHR-1210 plus apatinib as second-line therapy in patients with refractory or resistant SCLC (NCT03417895). This study is ongoing.

In conclusion, the findings of this study suggest that apatinib monotherapy has promising efficacy and acceptable toxicities in patients with recurrent ES-SCLC after second/third-line chemotherapy. Limitations of the study include its single-arm design, a selection bias due to the lack of a control group, a relatively small sample size and a high rate of dosage modifications. Nevertheless, these results, together with emerging evidences, provide a basis for future explorations of apatinib alone or in combination with novel therapeutic approaches such as immunotherapy in patients with relapsed ES-SCLC. Consequently, a well-designed phase III trial with a larger cohort is warranted.

## Supplementary information

supplementary files, tables, figures, information and legends

## Data Availability

All data included in this study are available upon request by contact with the corresponding author.
