# Supplementary material for: Apatinib in patients with extensive-stage small-cell lung cancer after second-line or third-line chemotherapy: a phase II, single-arm, multicentre, prospective study
Source: Br J Cancer. 2019 Sep 16;121(8):640–6. doi: 10.1038/s41416-019-0583-6 (PMC6889407; doi:10.1038/s41416-019-0583-6)

Supplementary Tables

Table 1

| **Reasons for dose reduction** | **Patients requiring apatinib dose reduction, *n* (%)** |
| --- | --- |
| Hand-foot syndrome | 11 (73**.**3%) |
| Hypertension | 10 (66**.**7%) |
| Proteinuria | 1 (6**.**7%) |
| Mucositis | 1 (6**.**7%) |
| Thrombocytopenia | 1 (6**.**7%) |
| Elevated aminotransferase | 1 (6**.**7%) |
| Fatigue | 1 (6**.**7%) |
| ***Table S1:* Reasons for apatinib dose reductions in 15 patients** | |

Table 2

| **Variable** | | **Univariate analysis** | | |  | | **Multivariate analysis** | | |
| --- | --- | --- | --- | --- | --- | --- | --- | --- | --- |
|  |  | **HR** | **95% CI** | ***p* value** | | **HR** | | **95% CI** | ***p* value** |
| **Age at diagnosis** | <65 years | 1**.**0 |  |  | |  | |  |  |
|  | ≥65 years | 1**.**3 | (0**.**6-2**.**8) | 0**.**507 | |  | |  |  |
| **Sex** | Male | 1**.**0 |  |  | |  | |  |  |
|  | Female | 0**.**8 | (0**.**2-2**.**7) | 0**.**730 | |  | |  |  |
| **ECOG PS** | 0 | 1**.**0 |  |  | |  | |  |  |
|  | 1 | 1**.**0 | (0**.**0-378**.**7) | 1**.**0 | |  | |  |  |
|  | 2 | 1.0 | (0.0-757903**.**1) | 1**.**0 | |  | |  |  |
| **Smoking history** | No | 1**.**0 |  |  | |  | |  |  |
|  | Yes | 1.2 | (0**.**4-3.6) | 0**.**690 | |  | |  |  |
| **Stage** | Limited |  |  |  | |  | |  |  |
|  | Extensive |  |  | NA | |  | |  |  |
| **Liver metastases** | No | 1.0 |  |  | | 1**.**0 | |  |  |
|  | Yes | 3**.**6 | (1.5-8**.**5) | **0.004** | | 3**.**9 | | (1**.**5-10**.**6) | **0.007** |
| **Brain metastases** | No | 1**.**0 |  |  | | 1**.**0 | |  |  |
|  | Yes | 0**.**9 | (0**.**4-2**.**1) | 0**.**841 | | 0**.**6 | | (0**.**2-1**.**5) | 0**.**258 |
| **Previous lines of treatment** | Second | 1**.**0 |  |  | | 1**.**0 | |  |  |
|  | Third | 0**.**7 | (0**.**3-1**.**6) | 0**.**434 | | 1.0 | | (0**.**4-2**.**5) | 0**.**996 |
| **Relapse type** | Refractory | 1.0 |  |  | |  | |  |  |
|  | Sensitive | 0.6 | (0**.**3-1**.**3) | 0.224 | |  | |  |  |
| **Platinum re-challenge in second-line treatment** | No | 1.0 |  |  | |  | |  |  |
|  | Yes | 0.6 | (0**.**3-1**.**3) | 0.202 | |  | |  |  |
| ***Table S2*: Univariate and multivariate Cox analysis for association of baseline characteristics and treatment-related variables with progression-free survival (*n*=38)** | | | | | | | | | |

Table 3

| **Variable** | | **Univariate analysis** | | |  | **Multivariate analysis** | | |
| --- | --- | --- | --- | --- | --- | --- | --- | --- |
|  |  | **HR** | **95% CI** | ***p* value** | | **HR** | **95% CI** | ***p* value** |
| **Age at diagnosis** | <65 years | 1**.**0 |  |  | |  |  |  |
|  | ≥65 years | 1**.**2 | (0**.**5-2**.**7) | 0**.**638 | |  |  |  |
| **Sex** | Male | 1**.**0 |  |  | |  |  |  |
|  | Female | 0**.**7 | (0**.**2-2**.**8) | 0**.**575 | |  |  |  |
| **ECOG PS** | 0 | 1**.**0 |  |  | |  |  |  |
|  | 1 | 1**.**0 | (0**.**3-3**.**9) | 1**.**000 | |  |  |  |
|  | 2 | 1**.**0 | (0**.**0-223355**.**5) | 1**.**000 | |  |  |  |
| **Smoking history** | No | 1**.**0 |  |  | |  |  |  |
|  | Yes | 2.0 | (0**.**5-8**.**6) | 0**.**332 | |  |  |  |
| **Stage** | Limited |  |  |  | |  |  |  |
|  | Extensive |  |  | NA | |  |  |  |
| **Liver metastases** | No | 1**.**0 |  |  | | 1**.**0 |  |  |
|  | Yes | 1**.**8 | (0**.**9-3**.**7) | 0**.**119 | | 1**.**7 | (0**.**7-4**.**2) | 0**.**232 |
| **Brain metastases** | No | 1**.**0 |  |  | | 1**.**0 |  |  |
|  | Yes | 1**.**2 | (0**.**5-2**.**7) | 0**.**713 | | 1.0 | (0**.**4-2**.**4) | 0**.**970 |
| **Previous lines of treatment** | Second | 1**.**0 |  |  | | 1**.**0 |  |  |
|  | Third | 0.6 | (0**.**3-1**.**4) | 0**.**227 | | 0**.**8 | (0**.**3-2**.**1) | 0**.**644 |
| **Relapse type** | Refractory | 1.0 |  |  | |  |  |  |
|  | Sensitive | 1.0 | (0**.**5-2.1) | 0.914 | |  |  |  |
| **Platinum re-challenge in second-line treatment** | No | 1.0 |  |  | |  |  |  |
|  | Yes | 1.1 | (0**.**5-2.5) | 0.747 | |  |  |  |
| ***Table S3*: Univariate and multivariate Cox analysis for association of baseline characteristics and treatment-related variables with overall survival (*n*=38)** | | | | | | | | |

Table 4

| **Variable** | | **Univariate analysis** | | |
| --- | --- | --- | --- | --- |
|  |  | **HR** | **95% CI** | ***p* value** |
| **Proteinuria** | No | 1**.**0 |  |  |
|  | Yes | 1.0 | (0**.**4-2**.**2) | 0**.**920 |
| **Fatigue** | No | 1**.**0 |  |  |
|  | Yes | 1**.**3 | (0**.**6-2**.**6) | 0**.**542 |
| **Hypertension** | No | 1**.**0 |  |  |
|  | Yes | 0**.**6 | (0**.**3-1.4) | 0**.**239 |
| **Mucositis** | No | 1**.**0 |  |  |
|  | Yes | 1**.**1 | (0**.**5-2**.**7) | 0**.**793 |
| **Hand-foot syndrome** | No | 1**.**0 |  |  |
|  | Yes | 1.0 | (0.4-2**.**1) | 0**.**915 |
| ***Table S4*: Univariate Cox analysis for association of the treatment-related adverse events with progression-free survival in patients with at least one post-baseline efficacy assessment (*n*=38)** | | | | |

**.**

Table 5

| **Variable** | | **Univariate analysis** | | |
| --- | --- | --- | --- | --- |
|  |  | **HR** | **95% CI** | ***p* value** |
| **Proteinuria** | No | 1**.**0 |  |  |
|  | Yes | 1**.**4 | (0**.**6-3**.**3) | 0**.**372 |
| **Fatigue** | No | 1**.**0 |  |  |
|  | Yes | 2.1 | (1.0-4**.**5) | 0**.**064 |
| **Hypertension** | No | 1**.**0 |  |  |
|  | Yes | 0.8 | (0**.**4-1**.**7) | 0**.**570 |
| **Mucositis** | No | 1**.**0 |  |  |
|  | Yes | 1**.**5 | (0**.**7-3**.**5) | 0**.**303 |
| **Hand-foot syndrome** | No | 1**.**0 |  |  |
|  | Yes | 1.0 | (0**.**5-2**.**1) | 0**.**987 |
| ***Table S5:* Univariate Cox analysis for the association of treatment-related adverse events with overall survival in patients with at least one post-baseline efficacy assessment (*n*=38)** | | | | |

Supplementary Figure 1

.
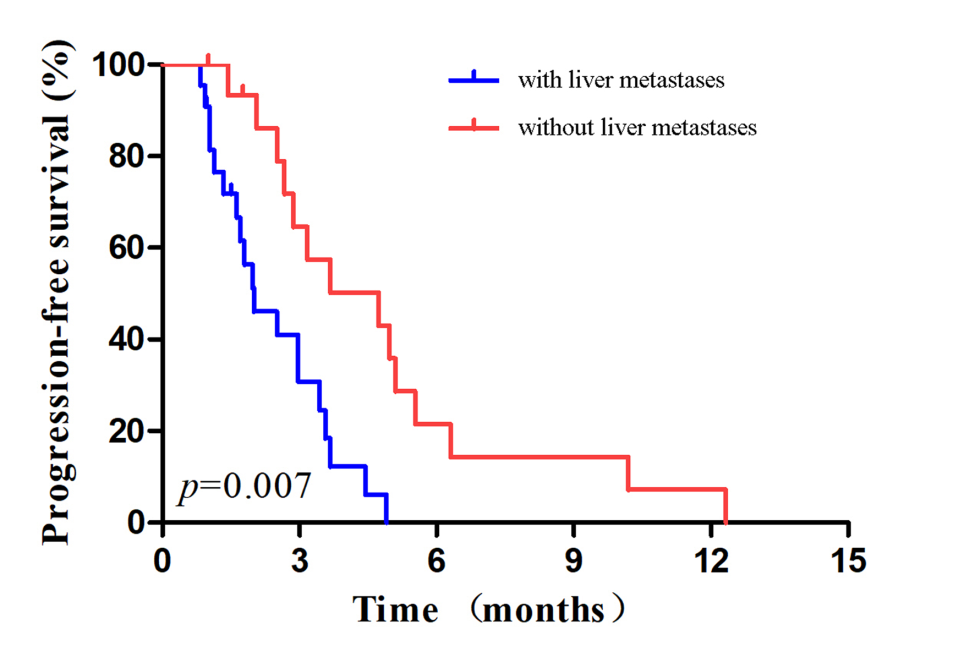


**Supplementary Figure 1: Kaplan-Meier estimates for progression-free survival by liver metastasis in patients with at least one post-baseline efficacy assessment (*n*=38).**

A single-arm multi-center phase II study of apatinib in patients with ES-SCLC after second/third-line chemotherapy

Principal Investigator：Yun Fan, Professor

Department of Chemotherapy, Zhejiang Cancer Hospital

Version：1.0

Date：July 10, 2016

**content**

[1.Introductions 9](#_Toc2196)

[2. Objectives 11](#_Toc29119)

[2.1 Primary Objective 11](#_Toc28614)

[2.2 Secondary Objectives 11](#_Toc20874)

[3.Study Design 11](#_Toc16226)

[3.1 Study Design 12](#_Toc2148)

[3.2 Sample Size 12](#_Toc20977)

[4. Subject Population 12](#_Toc13054)

[4.1 Inclusion Criteria 12](#_Toc5226)

[4.2 Exclusion Criteria 13](#_Toc26968)

[5. Treatment and Administration 15](#_Toc10795)

[6. Dose Modification 15](#_Toc26445)

[7. Study Procedures 15](#_Toc13770)

[7.1 Screening Phase 16](#_Toc27288)

[7.2 Treatment Phase 16](#_Toc5519)

[7.3 Post-treatment Phase 17](#_Toc31222)

[8. Efficacy 17](#_Toc6287)

[8.1 Evaluations 17](#_Toc9801)

[8.2 Endpoints 17](#_Toc6588)

[9. Safety Evaluations 18](#_Toc7662)

[9.1 Adverse Event 18](#_Toc6253)

[9.2 Serious Adverse Event 18](#_Toc27551)

[9.3Attribution Definitions 19](#_Toc25128)

[9.4 Safety Analyses 20](#_Toc17823)

[10. Statistical Analyses 20](#_Toc16308)

[10.1 Study Population Definitions 20](#_Toc14077)

[10.2 Analysis Method 20](#_Toc26552)

[11. Regulatory Ethics Compliance 21](#_Toc31792)

[11.1 Investigator Responsibilities 21](#_Toc19327)

[11.2 Informed Consent 21](#_Toc30046)

[11.3 Compensation to Research Subjects 22](#_Toc13966)

[11.4 Institutional Review Board (IRB) 22](#_Toc25607)

[12. Administrative Requirements 22](#_Toc16430)

1. **Introduction**

Lung cancer is the most common malignancy in the world, which was newly diagnosed in 733, 3 thousands Chinese in 2015 and responsible for 610.2 thousands cancer deaths (Chen et al., 2016). As a highly aggressive and lethal malignancy, small-cell lung cancer accounts for approximately 14% of newly diagnosed lung cancer cases. Almost two thirds of patients present with extensive disease (ED) at diagnosis (Jemal et al., 2008). Despite being strikingly sensitive to systemic chemotherapy resulting in dramatic responses, the majority of patients presents a high recurrence, and dies from systemic metastases (Simon et al., 2007). Combination of platinum and etoposide is the gold standard chemotherapy in current first-line treatment strategies (Stratigos et al., 2016). When the disease relapse, the standard second-line therapy such as irinotecan, topotecan et al only modestly improves outcomes and the OS (overall survival) for patients with ED-stage disease is less than one year (Ardizzoni et al., 1997; von Pawel et al., 1999). For the patients with extensive stage small lung cancer who experienced progression with two or more lines of chemotherapy, it still has no standard treatment strategies. The current chemotherapy strategies show limited response rate and unsatisfactory survival rates. During past decades, little progress has been made with no new drugs approved. Therefore, the development of more effective novel strategies is an unmet need for chemotherapy-refractory SCLC.

Angiogenesis is recognized as one of the major hallmark of tumor progression and metastasis , which is defined by Weinberg and Hanahan at the conceptual framework (Hanahan and Weinberg, 2011). In SCLC, angiogenesis also plays a key role not only in the formation of neovessels but also in the growth, invasion and metastases. It was reported that the VEGF levels was positively associated with microvessel density, which was confirmed as an independent prognostic factor in SCLC patients (Lucchi et al., 2002; Lund et al., 2000). As the core component of the angiogenesis, the VEGF pathway is active in SCLC. Salven et al. firstly described that the VEGF levels and tumor stage were the only two independent prognostic factors in untreated SCLC patients (Salven et al., 1998). Based on a systematic review and meta-analysis by Zhan et al., VEGF was confirmed as a prognostic factor in SCLC(Zhan et al., 2009) In addition, human SCLC cell lines express functional VEGFR2/3 (vascular endothelial growth factor receptor) and PDGFR-β (platelet-derived growth factor receptor) , indicating that VEGF/VEGFR is an autocrine growth regulator in SCLC (Ioannou et al., 2009; Tanno et al., 2004).

The inhibition of angiogenesis has shown modest efficacy in several solid human cancers, including non-small cell lung cancer (NSCLC). In SCLC, several anti-angiogenic agent such Bev (bevacizumab) (Horn et al., 2009; Petrioli et al., 2015; Spigel et al., 2009; Spigel et al., 2011), sunitinb (Ready et al., 2015; Schneider et al., 2011), thalidomide(Lee et al., 2009; Pujol et al., 2007), aflibercept (Allen et al., 2014), vandetanib (Arnold et al., 2007), cediranib (Ramalingam et al., 2010), sorafenib (Gitlitz et al., 2010), pazopanib (Rabasseda, 2015) and rovalpituzumab-tesirine (NCT02674568) were tested as the second line therapy strategy in clinical trials. Unfortunately, results were disappointing, and none of them has received regulatory approval caused by limited efficacy. Ji-Youn Han et al. conducted a Phase Ⅱ study on nintedanib, an oral multi-target tyrosine kinase inhibitor effectively against VEGFR-1/2/3, FGFR-1/2/3 and PDGFRα/β in SCLC patients who exhibited progression after one or two prior chemotherapy or chemo/radiotherapy. The ORR (objective response rate) was 5% and median PFS (progression free survival) and OS were 1.0 month and 9.8 month respectively. Thus, ninedanib only exhibited limited activity owing to unfulfilled criteria in relapsed or refractory SCLC (Stratigos et al., 2016).

Apatinib is an oral tyrosine kinase inhibitor specifically vascular endothelial growth factor receptor 2(VEGFR-2). In preclinical models, apatinib inhibited the proliferation, migration and tube formation of HUVEC (human umbilical vein endothelial cells) in vitro and blocked the rat aortic budding. In addition, apatinib presented a statistically significant inhibition of established human lung, colon and gastric tumor xenograft models (Han et al., 2016; Stratigos et al., 2016). Clinical activity of apatinib in gastric cancer (Li et al., 2016), breast cancer (both non-triple-negative breast cancer and triple-negative breast cancer)(Hu et al., 2014a; Hu et al., 2014b), non-squamous NSCLC and hepatocellular carcinoma were described in phage Ⅱ or Ⅲ studies. Given the promising activity through the inhibition of angiogenesis and an acceptable toxicity profile, we prospectively assessed the efficacy and safety of apatinib in patients with extensive-stage small lung cancers after the failure of second or third-line chemotherapy.

**2. Objectives**

**2.1 Primary Objective**

- **Objective response rate(ORR)**

Objective response rate defined as confirmed complete response or partial response under RECIST 1.1 criteria.

**2.2 Secondary Objectives**

- **Progression-free survival (PFS)**

Progression-free survival estimated using Kaplan-Meier methods is defined as the time from registration to the earlier of death or disease progression. Patients alive without disease progression are censored at the date of last disease evaluation.

- **Overall survival (OS)**

Overall survival defined as the time from registration to death for any reason (Patients lost are recorded as the date of last follow-up).

- **Disease control rate (DCR)**

Disease control rate is defined as the percentage of patients who achieved a complete response, a partial response or stable disease.

- **Complete response (CR):** the disappearance of all target lesions based on RECIST 1.1.
- **Partial response (PR):** at least a 30% decrease in the sum of diameters of target lesions, taking as reference the baseline sum of the diameters of target lesions based on RECIST 1.1.
- **Progressive disease (PD):** an increase of at least 20% in the sum of the diameters of target lesions, taking as reference the smallest sum of the diameters of target lesions recorded since the treatment started. Equivocal progression of non-target lesions also qualifies as PD.
- **Stable disease (SD):** any cases that do not qualify for either partial response or progressive disease.
- **Safety data**

Frequency and severity of adverse effects as defined by CTCAE version 4.03.

**3. Study Design**

**3.1 Study Design**

This is a phase II, single-arm, multi-center, open-label study.

**3.2 Sample Size**

This study followed Simon’s two-stage optimum design, with a type I error rate of 10% and a power of 80% to reject the null hypothesis. The previously reported objective response rate with topotecan monotherapy as second-line therapy in SCLC is 7%. In the present study, the primary expectation of an objective response with apatinib monotherapy was 19%. Consequently, 18 eligible patients received treatment in the first stage of the study with at least two responses required to continue the enrollment. In stage 2, 19 additional patients were enrolled for a total sample size of 37. Overall, if a total of 5 responses or more were observed, the treatment regimen would be considered successful. Assuming a 5 % missing follow-up rate for the subjects, a total sample size of 39 was required, this study design planned to enroll 40 patients.

**4. Subject Population**

**4.1** **Inclusion Criteria**

Patients must meet all of the following criteria to be eligible for the study.

- Able to provide written informed consent and can understand and comply with the requirements of the study and the schedule of assessments.
- Age ≥ 18 years on the day of signing the informed consent form (or the legal age of consent in the jurisdiction in which the study is taking place).
- Patients had histologically confirmed ES-SCLC.
- With disease progression (according to Response Evaluation Criteria in Solid Tumors [RECIST] version 1.1) following two or three previous chemotherapy regimens，including a platinum-based regimen.
- Eastern Cooperative Oncology Group (ECOG) performance status of 0-2.
- Life expectancy of at least 12 weeks.
- Patients with one or more measurable tumor lesion (according to Response Evaluation Criteria in Solid Tumors [RECIST] version 1.1). Previously irradiated lesions can only be considered as measurable disease if disease progression has been unequivocally documented at that site since radiation and the previously irradiated lesion is not the only site of disease.
- Patients with adequate bone marrow function and renal function (white blood cell count of ≥3.5×10^3^ cells/μL, absolute neutrophil count (ANC) of ≥1.5×10^3^ cells/μL, platelet count of ≥100×10^3^ cells/μL, and haemoglobin concentration of ≥9.0 g/dL), adequate hepatic function (aspartate transaminase [AST] and alanine transaminase [ALT] ≤ 1.5 × upper normal limit (UNL), bilirubin level ≤ 1.5 ×UNL) . Note: Patients must not have required a blood transfusion or growth factor support within the 14 days before sample collection.
- Patients can take oral medicine. Patients have the ability to understand and voluntarily sign the informed consent and allow adequate follow-up.
- Patients with a history of treated CNS metastases are eligible, provided they meet all of the following criteria:

Only supratentorial and cerebellar metastases allowed (i.e., no metastases to midbrain, pons, medulla or spinal cord);

No ongoing requirement for corticosteroids as therapy for CNS disease;

No whole-brain radiation within 14 days prior to randomization;

- Patients with new symptomatic CNS metastases detected at the screening scan must receive radiation therapy for CNS metastases. Following treatment, these patients may then be eligible without the need for an additional brain scan prior to randomization, if all other criteria are met.

**4.2 Exclusion Criteria**

Patients will be excluded from the study for any of the following reasons.

- Previous treatment with anti-angiogenesis drugs, including but not limited to Bevacizumab, Endostatin, Apatinib, etc before screening.
- Patients with toxicities (as a result of prior anticancer chemotherapy including radiation) which have not recovered to baseline or stabilized, except for AEs not constituting a likely safety risk (including but not limited to alopecia, rash, pigmentation, specific laboratory abnormalities etc).
- Received prior chemotherapy used to control cancer ≤ 28 days (or ≤ 5 half-lives, whichever is shorter) prior to randomization.
- Received any herbal medicine used to control cancer within 14 days prior to randomization.
- History of interstitial lung disease, non-infectious pneumonitis or severe clinical infection (> NCI-CTCAE version 4.0, infection standard II).
- History of cardiovascular disease: congestive heart failure (CHF) > New York Heart Association (NYHA) II, active coronary artery disease(patients with myocardial infarction six months ago can be recruited),Suffered from grade II or above myocardial ischemia or myocardial infarction, uncontrolled arrhythmias (including QT interval male ≥ 450 ms, female ≥ 470 ms). Grade III-IV cardiac insufficiency according to New York Heart Association (NYHA) criteria or echocardiography check: left ventricular ejection fraction (LVEF)<50%
- The patients previously received allogeneic organ transplantation. Bleeding tendency or coagulation disorders. Patients who need renal dialysis. Suffered from other tumor within 5 years (Except: cervical carcinoma in situ, cured basal cell carcinoma, cured bladder epithelial tumor).
- Uncontrolled hypertension (systolic pressure>150 mmHg, or diastolic pressure> 90 mmHg).
- Thrombosis or embolism (cerebrovascular accidents including transient ischemic attack within the last 6 months).
- Pulmonary hemorrhage > CTCAE grade 2 within 4 weeks before enrollment. Other organ hemorrhage > CTCAE grade 3 within 4 weeks before enrollment. Severe uncured wounds, ulcers or fracture, uncured dehydration.
- Factors influencing the safety and compliance of patients.
- Pregnant or breast-feeding.
- The researchers believe that the patient is not suitable to participate in the study.
- Patients with dysphagia.
- Major surgical procedure requiring general anesthesia, or significant traumatic injury ≤ 28 days prior to randomization, or anticipation of need for major surgical procedure during the course of the study.
- Concurrent participation in another therapeutic clinical study.
- Underlying medical conditions (including laboratory abnormalities) or alcohol or drug abuse or dependence that will be unfavorable for the administration of study drug or affect the explanation of drug toxicity or AEs or result in insufficient or impaired compliance with study conduct.
- Untreated, uncontrolled and symptomatic CNS metastases:

• Patients with a history of treated and, at the time of screening, asymptomatic CNS metastases are eligible, provided they meet all the following:

- Brain imaging at screening shows no evidence of interim progression

- Have measurable disease outside the CNS, only supratentorial metastases allowed

- No ongoing requirement for corticosteroids as therapy for CNS disease;

- No whole brain radiation within 14 days prior to randomization

• Patients with new asymptomatic CNS metastases detected at the screening scan must receive radiation therapy for CNS metastases.

- Following treatment, these patients may then be eligible, provided all other criteria.

**5. Treatment and Administration**

Apatinib, 500 mg, administered orally once daily. One treatment cycle was 30 days long. Treatment was continued until disease progression, patient withdrawal, unacceptable toxicity or death.

**6. Dose Modification**

Dose interruptions or reductions were permitted for management of adverse events (AEs), but only one dose reduction per patient was allowed (250 mg QD) and dose re-escalation was not permitted. When ≥ grade 3 haematological or ≥ grade 2 non-haematological toxicities or a clinically intolerable grade 2 adverse event (AE) occurred at any time, dose interruptions or reductions were allowed. In such cases, treatment was allowed to delay until recovery to ≤ grade 2 haematological or ≤ grade 1 non-haematological toxicity and was then resumed at a reduced dosage of 250 mg once daily. Repeated dose interruptions were allowed for a maximum of 14 days on each occasion. Once dose interruption had occurred, apatinib was resumed at a reduced dosage for subsequent treatment.

**7. Study Procedures**

**7.1 Screening Phase**

Screening procedures to be completed within 28 days before treatment:

- Signed informed consent
- Review of eligibility criteria
- Review of medical history and demographics
- Physical examination
- Vital signs
- Eastern Cooperative Oncology Group (ECOG) performance status
- Laboratory tests including hematology and serum chemistry, and urine routine.
- Tumor assessments, including radiographic imaging of the chest, abdomen and pelvis by computed tomography (CT) or magnetic resonance imaging (MRI).

**7.2 Treatment Phase**

During the treatment of apatinib, patients had to come to outpatient clinic every 30 days (± 7 days).

- Physical examination, Vital signs, Eastern Cooperative Oncology Group (ECOG) performance status and laboratory tests including hematology and serum chemistry, and urine routine every 30 days (± 7 days). Urinary protein quantity in 24 hours will be tested if urinary protein is 2+.
- Tumour assessments were conducted by radiographic imaging [computed tomography (CT) and magnetic resonance imaging (MRI)] at baseline, after the first cycle of apatinib treatment, and then every two months (± 7 days) thereafter until disease progression occurred (investigator-assessed per RECIST, version 1.1) or treatment was discontinued. Once a partial response was occurred of apatinib treatment, another CT scan will be added one month later to confirm the partial response. If clinical symptoms of patients aggravate, we will consider taking CT scan ahead of time. Baseline tumour assessments included at least enhanced CT of the chest, abdomen, and pelvis, and enhanced MRI of the brain. Repeated radiographic imaging included enhanced CT of the chest and abdomen, and enhanced MRI of the brain in cases of symptom occurrence. All known sites of disease must be documented at screening and reassessed at each subsequent tumor evaluation. The same radiographic procedure used to assess disease sites at screening should be used throughout the study. In case of clinical progression, the date of progression will be defined as the date of the first imaging study that documents progression.
- Adverse events will be reported by the patients and be followed by the investigator until resolution or until a clinically stable endpoint is reached.

**7.3 Post-treatment Phase**

The subjects will continue on the schedule until: 1) radiographic disease progression, 2) the subject withdraws consent, 3) the subject begins subsequent anticancer therapy, or 4) the study is terminated. Once the subject discontinues the treatment, drug-related adverse events during treatment or within 30 days of last dose of the study should be recorded.

**8. Efficacy**

**8.1 Evaluations**

Measurable disease and the response criteria used in this protocol are defined in the RECIST guidelines (version 1.1) (attachment 1) and will be based on radiologic assessment only.

Appropriate radiological disease assessments (CT scans or MRI) will be performed before treatment, and should include, at a minimum, imaging of the chest, abdomen, and pelvis.

Disease will be assessed using CT scans or MRI at baseline and every 2 months during treatment until disease progression (investigator-assessed per RECIST version 1.1) or treatment discontinuation.

**8.2 Endpoints**

**Primary endpoints**

Objective response rate defined as confirmed complete response or partial response under RECIST 1.1 criteria.

**Secondary endpoints**

- **Safety**
- Progression-free survival estimated using Kaplan-Meier methods is defined as from assignment to the first time of disease progression or death from any cause, whichever comes first patients alive without disease progression are censored at the date of last disease evaluation.
- Overall survival defined as the time from registration to death for any reason (Patients lost to follow-up are recorded as the date of last follow-up).
- Disease control rate is defined as the percentage of patients who achieved a complete response, a partial response or stable disease.

**9. Safety Evaluations**

**9.1 Adverse Event**

**9.1.1 Definition of adverse events**

An adverse event is any untoward medical occurrence in a clinical study subject administered a medicinal product. An adverse event does not necessarily have a causal relationship with the treatment. An adverse event can therefore be any unfavorable and unintended sign, symptom, or disease temporally associated with the use of a medicinal product, whether or not related to that medicinal product.

This includes any occurrence that is new in onset or aggravated in severity or frequency from the baseline condition, or abnormal results of diagnostic procedures, including laboratory test abnormalities.

**9.1.2 Criterion for severity of adverse events**

Adverse reactions were graded 0-IV according to the National Cancer Institute Common Terminology Criteria for Adverse Events (NCI-CTCAE version 4.03) for Acute and Subacute Toxic and Side Reactions of Anticancer Drugs.

**9.2 Serious Adverse Event**

**9.2.1 Definition of adverse events**

A Serious Adverse Event (SAE) is defined as any adverse drug event (experience) occurring at any dose, including the following unexpected medical events:

- Results in death
- Is life-threatening
- Requires inpatient hospitalization or prolongation of existing hospitalization
- Results in persistent or significant disability/incapacity
- Is a congenital anomaly/birth defect
- Important Medical Event (IME) that may not result in death, be life threatening, or require hospitalization may be considered a serious adverse drug experience when, based upon medical judgment, they may jeopardize the patient or subject and may require medical or surgical intervention to prevent one of the outcomes listed in this definition.

All SAEs occurring during the study must be reported to Institutional Review Board (IRB) with 24 hours of their knowledge of the event. The initial and follow-up reports of a SAE should be made.

**9.2.2 Pregnancy**

Pregnancy during study should be reported as serious adverse events.

**9.2.3 Disease progression**

Disease progression (including symptoms and signs of progression) should not be reported as serious adverse events, but death due to disease progression should be reported as serious adverse events during the study or safety reporting period. Hospitalization due to symptoms and signs of disease progression should not be reported as serious adverse events. During the test or safety reporting period, if the final outcome of cancer is death, the events leading to death must be reported as serious adverse events.

**9.3 Attribution Definitions**

An adverse event is considered associated with the use of the drug if the attribution is possible, probable, or very likely by the definitions.

• **Not Related**

An adverse event is not related to the use of the drug.

**• Doubtful**

An adverse event for which an alternative explanation is more likely, e.g., concomitant drug(s), concomitant disease(s), or the relationship in time suggests that a causal relationship is unlikely.

**• Possible**

An adverse event might be due to the use of the drug. An alternative explanation, e.g., concomitant drug(s), concomitant disease(s), is inconclusive. The relationship in time is reasonable; therefore, the causal relationship cannot be excluded.

**• Probable**

An adverse event might be due to the use of the drug. The relationship in time is suggestive (e.g., confirmed by dechallenge). An alternative explanation is less likely, e.g., concomitant drug(s), concomitant disease(s).

**• Very likely**

An adverse event that is listed as a possible adverse reaction and cannot be reasonably explained by an alternative explanation, e.g., concomitant drug(s), concomitant disease(s).

The relationship in time is very suggestive.

**9.4 Safety Analyses**

All subjects who have received at least 1 dose of study drug will be included in the safety analysis. All the adverse events should be recorded in CRF and will be graded according to the National Cancer Institute Common Terminology Criteria for Adverse Events (NCI-CTCAE version 4.03) for Acute and Subacute Toxic and Side Reactions of Anticancer Drugs. Serious adverse and deaths will be listed. All adverse events resulting in discontinuation of study treatment, dose modification, the interruption of dosing, or a delay in treatment with the study drug will be noted.

**10. Statistical Analyses**

**10.1 Study Population Definitions**

In this study the following three populations will be defined for the analysis.

- Full Analysis Set (FAS): FAS comprised all enrolled patients who received at least one dose of the study medication according to the principle of intentional analysis (ITT). For cases that failed to observe all treatment procedures, the final outcome was carried forward with the last observation (LOCF).
- Per Protocol Set (PPS): PPS referred to a subset of the patients in FAS who were compliant with the protocol and without any major protocol violations including the violation of entry criteria.
- Safety Analysis Set (SAS): SAS included all enrolled patients who received at least one dose of study medication, but not those without any safety data.

**10.2 Analysis Method**

Subjects’ baseline characteristics will be summarized for the FAS. The efficacy analysis will be performed on both FAS and PPS. SAS will be performed on safety population.

Efficacy analysis: Objective response and disease control rate were evaluated by a binomial response rate and corresponding two-sided 95% exact CIs using the Clopper-Pearson method. Progression-free survival and overall survival were summarized descriptively using the Kaplan-Meier method; median values were estimated with two-sided 95% CIs, calculated using the Brookmeyer-Crowley method. Cox proportional hazards regression models were used for univariate and multivariate analysis to identify the potential factors associated with efficacy outcome.

Safety analysis: The safety parameters to be evaluated are the incidence, intensity, and type of adverse events, and clinical laboratory results by descriptive statistics.

A *p* value (two-sided) <0.05 was considered statistically significant. All analyses were performed with SPSS 22.0.

**11. Regulatory Ethics Compliance**

**11.1 Investigator Responsibilities**

The investigator is responsible for ensuring that the clinical study is performed in accordance with the protocol, current ICH guideline on Good Clinical Practice (GCP), and applicable regulatory and country-specific requirements.

**11.2 Informed Consent**

Each subject must give written consent according to local requirements after the nature of the study has been fully explained. The informed consent should be in accordance with principles that originated in the Declaration of Helsinki, current ICH and GCP guidelines, applicable regulatory requirements.

Before enrollment in the study, the investigator must explain to potential subjects the aims, methods, reasonably anticipated benefits, and potential hazards of the study, any discomfort participation in the study may entail. Subjects will be informed that their participation is voluntary and that they may withdraw consent to participate at any time. They will be informed that choosing not to participate will not affect the care the subject will receive for the treatment. The subject will be given the opportunity to ask questions.

After the explanation and before entry into the study, consent should be recorded by the subject’s personally dated signature. After having obtained the consent, a copy of the ICF must be given to the subject.

**11.3 Compensation to Research Subjects**

A subject is entitled to compensation if injury or death is due to adverse effect of investigational products. Compensation must be consistent with the laws, regulations, and guidelines of the region in which the study is conducted.

**11.4 Institutional Review Board (IRB)**

Before the start of the study, the investigator will provide the IRB with current and complete copies of the documents, which include, but are not limited to, final protocol, informed consent, investigators’ curriculum vitae, information regarding funding, and other potential conflicts of interest. The study will be undertaken only after the IRB has given full approval of all the documents. All the protocol amendments must be submitted to the IRB for review and approval before implementation of the changes.

**12. Administrative Requirements**

The investigators should performed the following aspects, which include, but are not limited to, protocol amendments, regulatory documentation, case report form completion, record retention, monitoring, and data quality control.


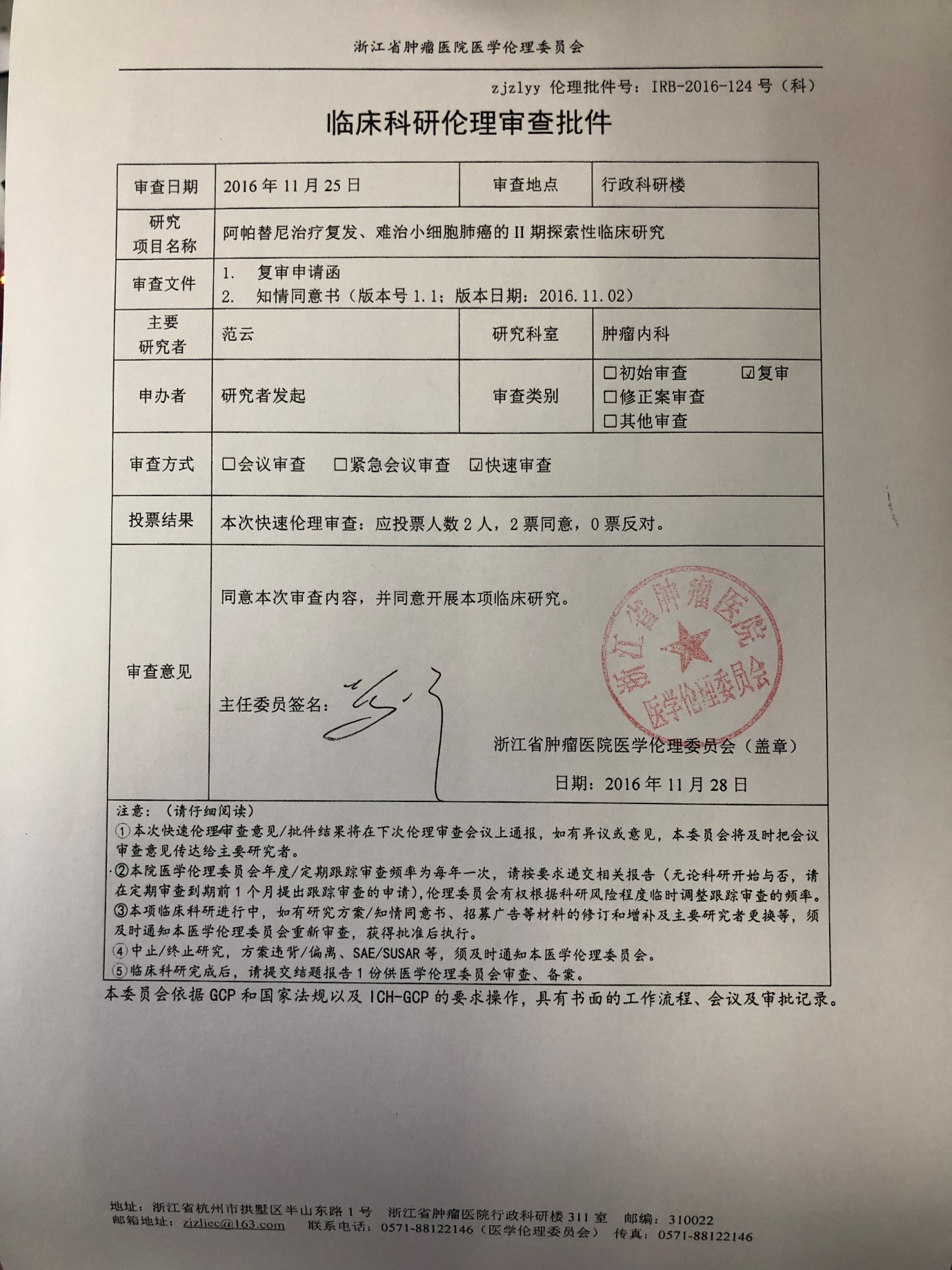

Supplement: Supplementary file 1 — supplementary files, tables, figures, information and legends [file 41416_2019_583_MOESM1_ESM.docx]
